# The Impact of Creatinine Clearance Rate, Daily Urinary Albumin, and Their Joint Effect on Predicting Death in Diabetic Inpatients After Discharge: An Observational Study

**DOI:** 10.1097/MD.0000000000002804

**Published:** 2016-02-12

**Authors:** I-Te Lee, Wayne H-H Sheu, Shih-Yi Lin

**Affiliations:** From the Department of Internal Medicine, Division of Endocrinology and Metabolism, Taichung Veterans General Hospital (ITL, WHHS, SYL); Chung Shan Medical University, Taichung (ITL, WHHS); School of Medicine, National Yang-Ming University, Taipei (ITL, WHHS); and Center for Geriatrics and Gerontology Taichung Veterans General Hospital (SYL), Taichung, Taiwan..

## Abstract

Renal clearance function and urinary albumin excretion are important markers for diabetic nephropathy. We assessed whether the creatinine clearance rate (CCR) and daily urinary albumin (DUA) excretion, which both require 24-hour urine data, are better predictors of mortality in diabetic inpatients compared with the estimated glomerular filtration rate (eGFR) and urine albumin–creatinine ratio (ACR).

We enrolled 1011 patients who were hospitalized due to poor glucose control, and collected clinical information, including 24-hour urine data, from their medical records. We determined the mortality rate after discharge by examining the national registry data in Taiwan.

The subjects had a median follow-up of 6.5 years (interquartile range between 3.5 and 9.6 years). Subjects with a CCR < 60 mL/min and a DUA ≥ 300 mg/d had the highest mortality rate, with a hazard ratio of 3.373 (95% confidence interval = 2.469–4.609), compared with the mortality rate in subjects with a CCR ≥ 60 mL/min and a DUA < 300 mg/d. In terms of predicting mortality in diabetic inpatients, ACR had a similar sensitivity to DUA (40.3% versus 38.0%), but eGFR provided lower sensitivity than CCR (54.5% versus 66.5%).

Creatinine clearance rate and DUA have an additive effect on predicting mortality in diabetic inpatients after discharge. Moreover, CCR is a more sensitive predictor of mortality than eGFR. Therefore, determining CCR using 24-hour urine data, as well as either ACR or DUA, should provide better prediction of mortality in diabetic nephropathy patients.

## INTRODUCTION

Diabetes mellitus is the leading cause of chronic kidney disease (CKD), which is associated with a high mortality rate and remains a global health burden.^1–4^ Although a decrease in renal clearance function and an increase in urinary albumin excretion have both been identified as important indicators for diabetic nephropathy,^5^ accurate early predictors of mortality for inpatients with diabetic nephropathy remain under investigation.

To assess renal clearance function and the stage of CKD, the creatinine clearance rate (CCR) is often used as a surrogate marker; however, it requires collection of 24-hour urine, which is inconvenient for clinical practice especially for outpatients.^6^ Therefore, several creatinine-based equations have been developed that only require spot urine data, including the estimated glomerular filtration rate (eGFR) derived from the Modification of Diet in Renal Disease (MDRD) equation.^7–9^ Indeed, the eGFR is well associated with renal events and mortality, and has become widely adopted in clinical practice. However, as there is some disagreement between CCR and eGFR in inpatients, CCR and eGFR should not be used interchangeably in clinical practice.^10^

Daily urinary albumin (DUA) excretion is also used as a predictive factor for renal events and mortality in diabetic patients.^11,12^ However, as the DUA also requires 24-hour urine data, the albumin to creatinine ratio (ACR), which is calculated from the concentrations of albumin and creatinine in a spot urine sample, is often used for outpatients. Indeed, the ACR has been validated for estimating urine albumin excretion.^6,9^

It has been reported that albuminuria and low eGFR are independent risk factors for all-cause mortality in diabetic population.^13–15^ As diabetic patients have a high prevalence of hospital admission,^16^ and it is practicable to collect the 24-hour urine data for diabetic patients when they are hospitalized, we were in an ideal position to examine the predictive value of CCR and DUA. In the present study, we aimed to examine whether 24-hour urine data (CCR and DUA) is more informative in predicting mortality than using the estimated formula (eGFR and ACR) for diabetic nephropathy. Furthermore, we assessed the mortality rate among different nephropathy conditions based on CCR and DUA in diabetic inpatients.

## METHODS

### Patients

The cohort study comprised diabetic subjects who were hospitalized in the Endocrinology and Metabolism ward in Taichung Veterans General Hospital due to a primary diagnosis of poor glucose control between August 1996 and August 2007. The clinical data were obtained by reviewing medical records. We also collected the 24-hour urine data, including urine volume and concentrations of albumin and creatinine. We only collected the record of the last admission with completed 24-hour urine data in the repeatedly hospitalized patients. Hypertension was defined as the use of antihypertensive medications or blood pressure higher than 130/80 mm Hg. Mortality data between August 1996 and December 2011 were obtained from the Collaboration Center of Health Information Application, Department of Health, Executive Yuan, Taiwan. Cardiovascular disease was defined as coronary heart disease, cerebrovascular disease or peripheral artery disease. The study complies with the Declaration of Helsinki and the research protocol was approved by the Institutional Review Board of Taichung Veterans General Hospital.

### Assessments

The biochemistry analyses were performed using blood samples collected after overnight fasting, and urinary samples were collected for 24 hours based on the standard procedure in our ward. Briefly, the collection of urine sample was started after first emptying the bladder at 7  am in the morning, and the collection was not finished until the bladder was emptied at 7  am the next morning. Serum creatinine was also detected during the collection of urine. Serum concentrations of total cholesterol, high-density lipoprotein cholesterol and, triglycerides were determined using enzymatic methods (Advia 1800; Siemens, New York, NY). Creatinine was determined using the Jaffé method (Advia 1800; Siemens). Urinary albumin was determined using the polyethylene glycol enhanced immunoturbidimetric method (Advia 1800; Siemens), and glycated hemoglobin was determined by cation-exchange high performance liquid chromatography (NGSP certificated, G8, Tosoh, Tokyo, Japan).

Creatinine clearance rate (mL/min) was determined from the following formula: (urine creatinine [mg/dL] × urine volume [mL])/(serum creatinine [mg/dL] × 1440 [minutes]). Daily urinary albumin (mg/d) was calculated by: urine albumin concentration (mg/dL) × urine volume (mL)/100.^5^ The eGFR (mL/min/1.73 m^2^) was calculated based on the MDRD equation using the following formula: eGFR (mL/min/1.73 m^2^) = 186 × (serum creatinine [mg/dL])^−1.154^ × (age [year])^ −0.203^ (×0.742, if female).^9^ Albumin–creatinine ratio was determined from the following formula: albumin (mg)/creatinine (g). Chronic kidney disease was defined as a CCR < 60 mL/d or an eGFR < 60 mL/min/1.73 m^2^; and albuminuria was defined as a DUA ≥ 300 mg/24 hours or ACR ≥ 300.^5,9^

### Statistical Analysis

All descriptive data are presented as mean ± standard deviation. Independent *t* test was used to compare continuous variables at baseline between 2 groups. χ^2^ test was used to compare categorical variables across groups. Receiver operating characteristic analysis was assessed for differentiating mortality between CCR and eGFR or DUA and ACR. Correlation coefficient was determined by Pearson correlation. Agreement between CCR and eGFR or DUA and ACR were assessed by the Bland–Altman plot analysis.^17^ Multivariate linear regression analyses were used to assess the risk factors for the patient with a higher eGFR than CCR. One-way analysis of variance was used to compare among all groups, and pairwise multiple comparisons were used to assess the differences between 2 groups if analysis of variance revealed the statistical significance. Univariate survival analysis was assessed by Kaplan–Meier analysis, and the overall significance was detected by the log-rank test. Multivariate Cox proportional hazards regression analyses were used to determine mortality according to CKD and albuminuria. Statistical analysis was performed using SPSS 22.0 (IBM, Armonk, NY).

## RESULTS

**FIGURE 1:**
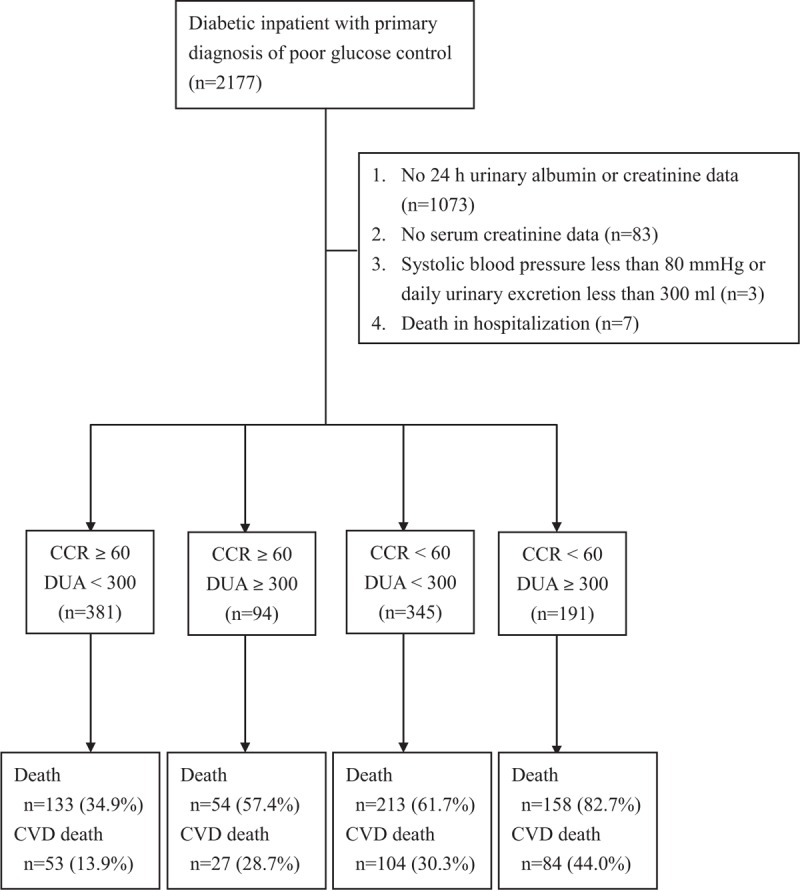
Flow diagram of enrolment of study subjects.

Among 2177 diabetic patients hospitalized due to poor glucose control during the study period, we enrolled 1011 subjects for analyses, after excluding subjects with incomplete urine data, a lack of serum creatinine, low blood pressure, a urine volume less than 300 mL/d, and death in hospitalization. There were 558 (55.2%) subjects who died during the 15 years (median of 6.5 years, interquartile range between 3.5 and 9.6 years) of follow-up (Figure 1). The baseline characteristics of the enrolled subjects are shown in Table 1.

**TABLE 1:**
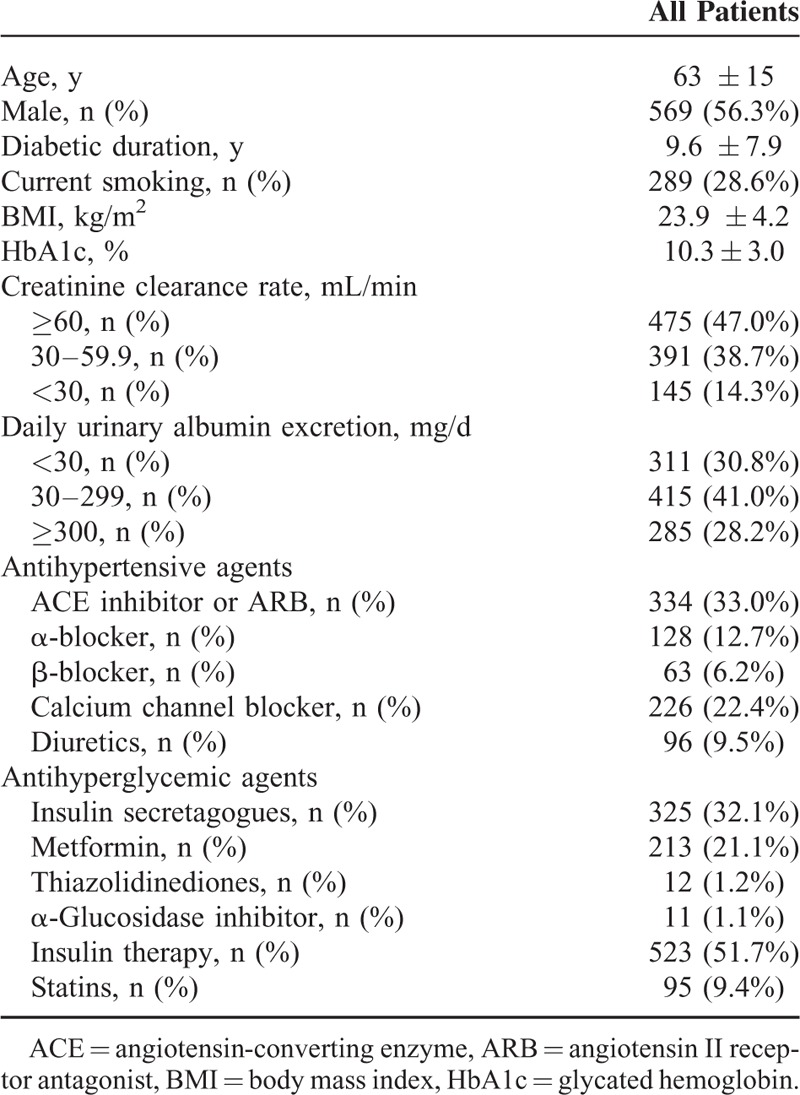
The Clinical Data of All Enrolled Subjects

There was a significantly positive correlation between ACR and DUA (*r* = 0.722, *P* < 0.00001), and the mean of differences between the values of ACR and DUA was 91.5 with 95% confidence interval (95% CI) for the bias ranging from −5.2 and 188.3 in the Bland–Altman plot. Although the correlation between eGFR and CCR was also significantly positive (*r* = 0.705, *P* < 0.00001), the mean of differences between the values of eGFR and CCR was 4.2 with 95% CI for the bias ranging from 2.5 and 5.8.

**FIGURE 2:**
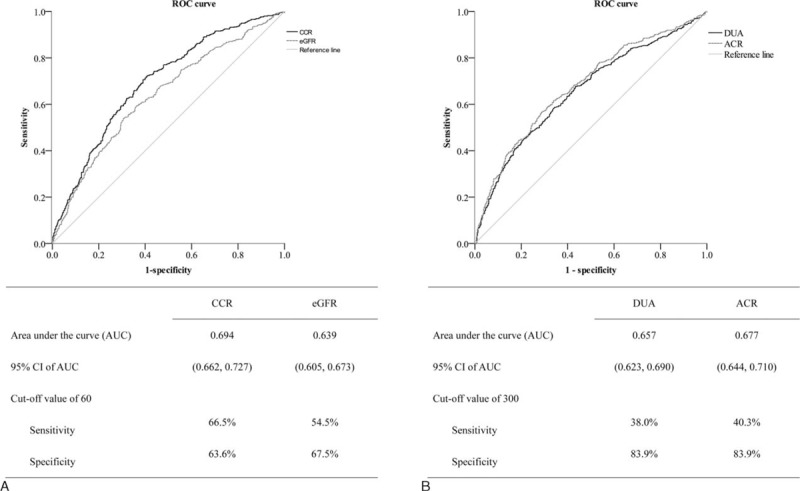
Receiver operating characteristic analysis curves for differentiating mortality based on (A) creatinine clearance rate and estimated glomerular filtration rate; or (B) daily urinary albumin excretion and urine albumin creatinine ratio.

Using ROC analyses for differentiating mortality based on CCR, the area under the curve (AUC) was 0.694 (95% CI = 0.662–0.727; Figure 2A). This was significantly greater (*P* < 0.001) than the AUC obtained using eGFR (0.639, 95% CI = 0.605–0.673). Using a CCR cutoff value of 60 mL/min had a higher sensitivity (66.5%) for predicting mortality than using eGFR (54.5%), in spite of a lower specificity (63.6%) than using eGFR (67.5%). On the other hand, the AUC (0.657, 95% CI = 0.623–0.690) for differentiating mortality based on DUA was similar to that (0.677, 95% CI = 0.644–0.710) obtained using ACR (Figure 2B).

To understand why the CCR and eGFR values were different in the diabetic inpatients, we divided the subjects into 2 groups, a CCR < eGFR (n = 562) group and a CCR ≥ eGFR (n = 449) group, and evaluated their clinical characteristics. We found that patients in the CCR < eGFR group were nonsmokers, and showed female predominance, lower body mass index (BMI), lower blood pressure, lower serum creatinine, lower triglycerides, and higher high-density lipoprotein cholesterol than those in the CCR ≥ eGFR group (Table 2). Using multivariate linear regression analyses, we determined that female gender, low BMI, low blood pressure, and low serum creatinine contributed to the overestimation of renal function using the MDRD eGFR formula. In particular, low BMI was an independent factor for overestimating renal function by the eGFR formula in both male and female patients (Table 3).

**TABLE 2:**
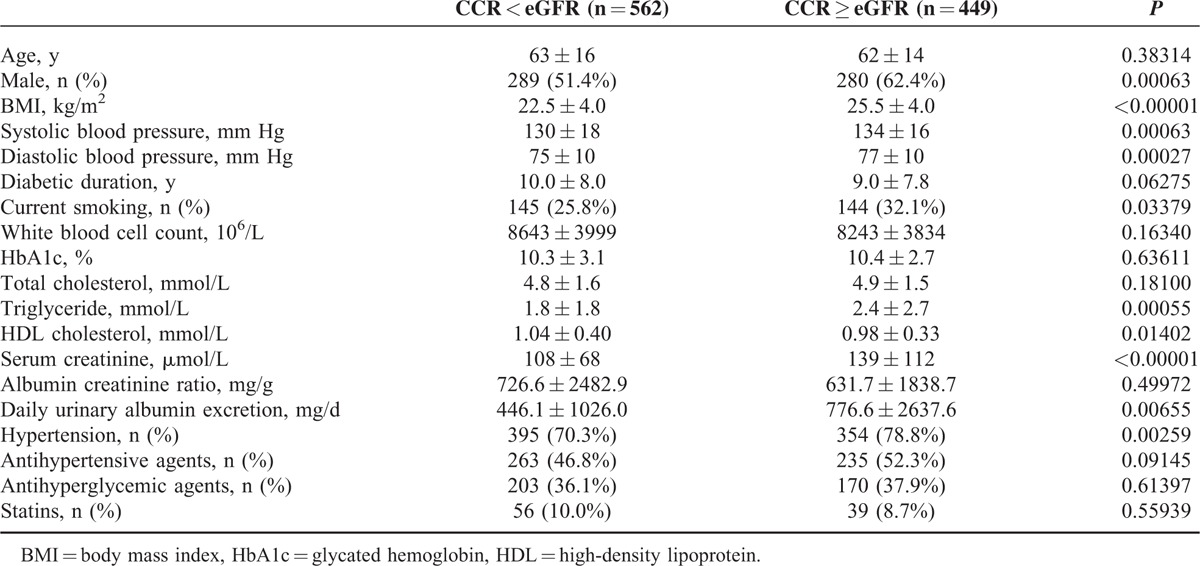
The Clinical Data of Subjects Grouped According to Differences in Creatinine Clearance Rate (Creatinine Clearance Rate, mL/min) and Estimated Glomerular Filtration Rate (mL/min/1.73 m^2^)

**TABLE 3:**
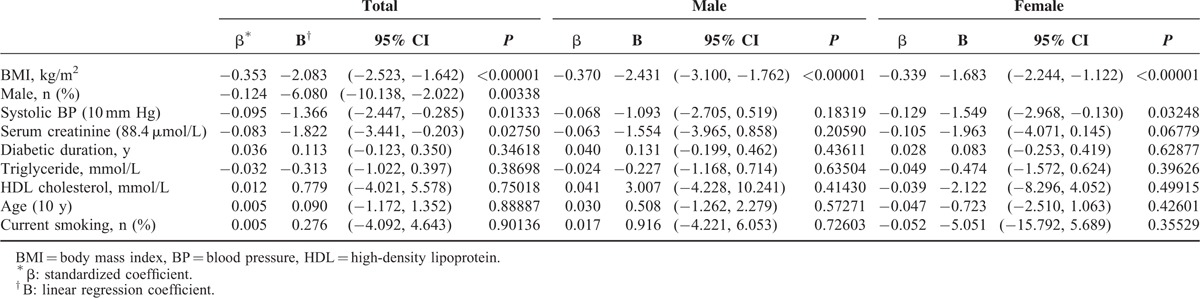
Multivariate Regression Analysis Showing the Independent Factors Contributing to the Overestimated Renal Function Using the Estimated Glomerular Filtration Rate Formula, Which Is Based on the Modification of Diet in Renal Disease Equation

**FIGURE 3:**
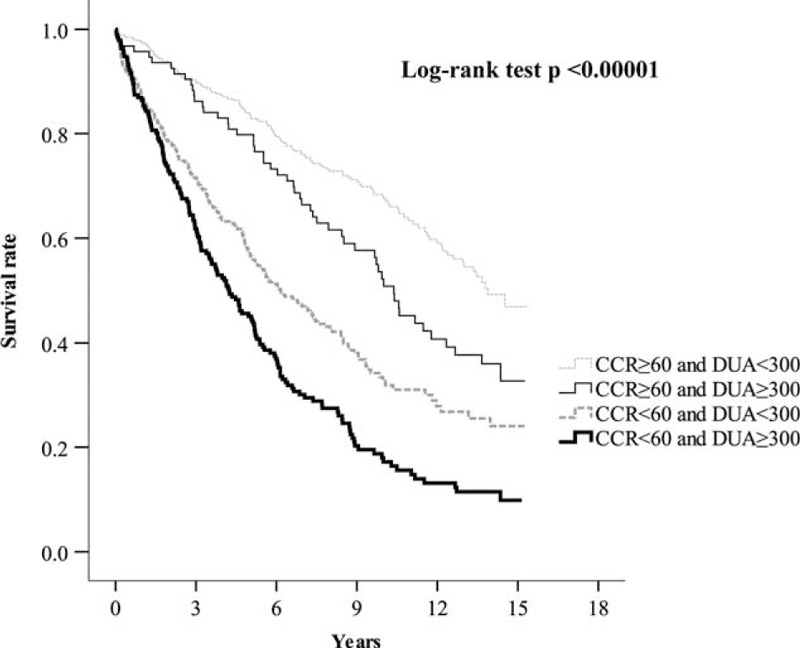
Kaplan–Meier curves showing survival rates grouped based on chronic kidney disease stage and albuminuria (ie, according to creatinine clearance rates, creatinine clearance rate, in mL/min and daily urine albumin excretion, daily urinary albumin, in mg/d).

Based on CKD stage (CCR, mL/min) and albuminuria (DUA, mg/d), we categorized all enrolled subjects into 4 groups: 381 subjects with CCR ≥ 60 mL/min and DUA < 300 mg/d, 94 subjects with CCR ≥ 60 mL/min and DUA ≥ 300 mg/d, 345 subjects with CCR < 60 mL/min and DUA < 300 mg/d, and 191 subjects with CCR < 60 mL/min and DUA ≥ 300 mg/d. The baseline clinical data for the patients in these 4 groups are shown in Table 4. During follow-up, 133 (34.9%), 54 (57.4%), 213 (61.7%), and 158 (82.7%) subjects in each group died, respectively. The mortality rate was significantly different (*P* [log-rank test] <0.00001) among the 4 groups based on CCR and DUA (Figure 3). Using Cox regression analysis, the patients with a CCR < 60 mL/min and DUA ≥ 300 mg/d had the highest hazard ratio (HR) for total mortality compared with patients with a CCR ≥ 60 mL/min and DUA < 300 mg/d (HR = 3.373 [95% CI = 2.469–4.609, *P* < 0.00001]; Table 5). Patients with a CCR < 60 mL/min and DUA < 300 mg/d or with a CCR ≥ 60 mL/min and DUA ≥ 300 mg/d also showed higher mortality rates compared with the patients with a CCR ≥ 60 mL/min and DUA < 300 mg/d (HR = 1.887 [95% CI = 1.428–2.494, *P* < 0.00001] and HR = 1.677 [95% CI = 1.124–2.503, *P* < 0.01137; respectively). Furthermore, the patients with a CCR < 60 mL/min and DUA ≥ 300 mg/d also had the highest HR for cardiovascular mortality when compared with patients with a CCR ≥ 60 mL/min and DUA < 300 mg/d (HR = 4.174 [95% CI = 2.589–6.730, *P* < 0.00001]; Table 6).

**TABLE 4:**
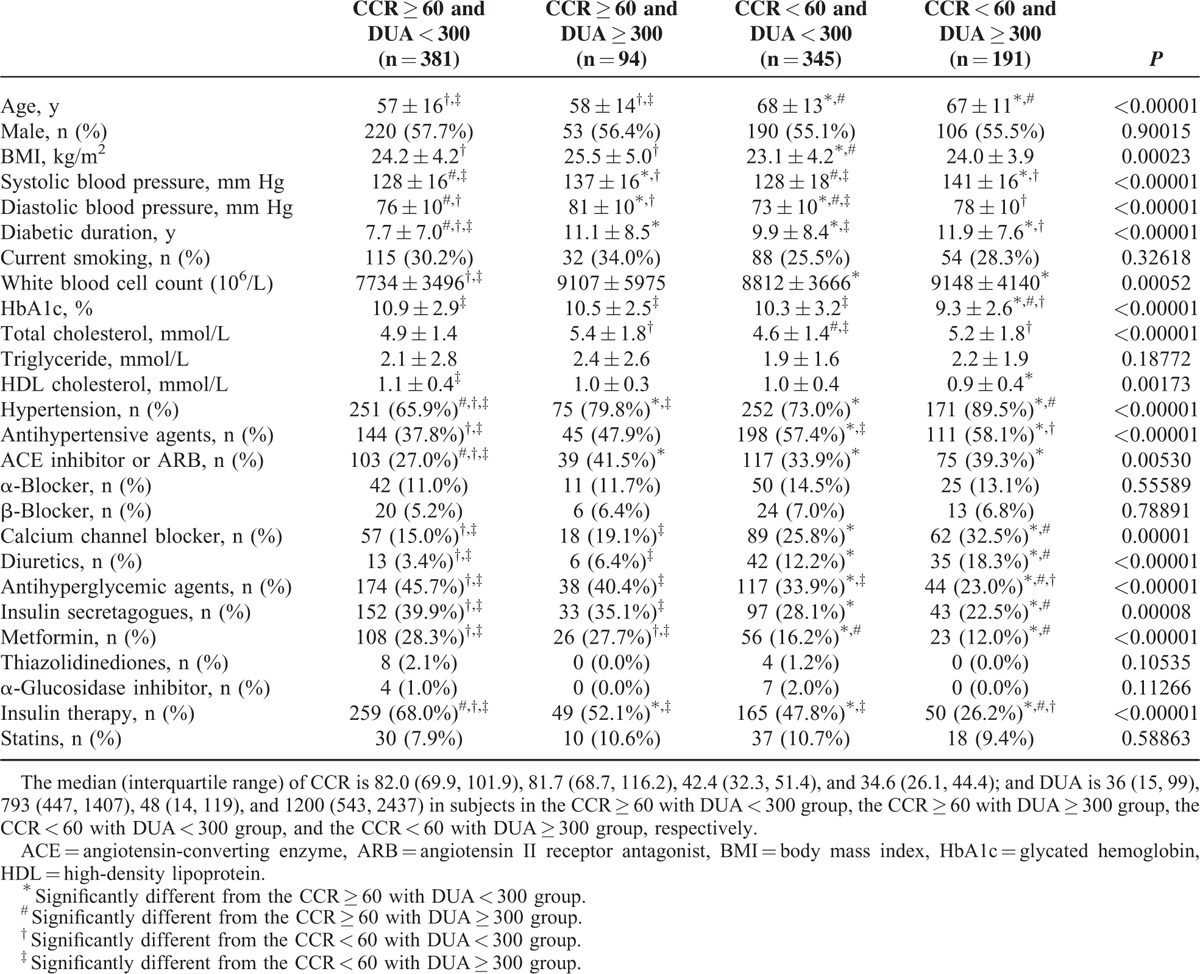
The Clinical Data of Subjects Grouped by Creatinine Clearance Rate (mg/mL) and Daily Urinary Albumin Excretion (mg/d)

**TABLE 5:**
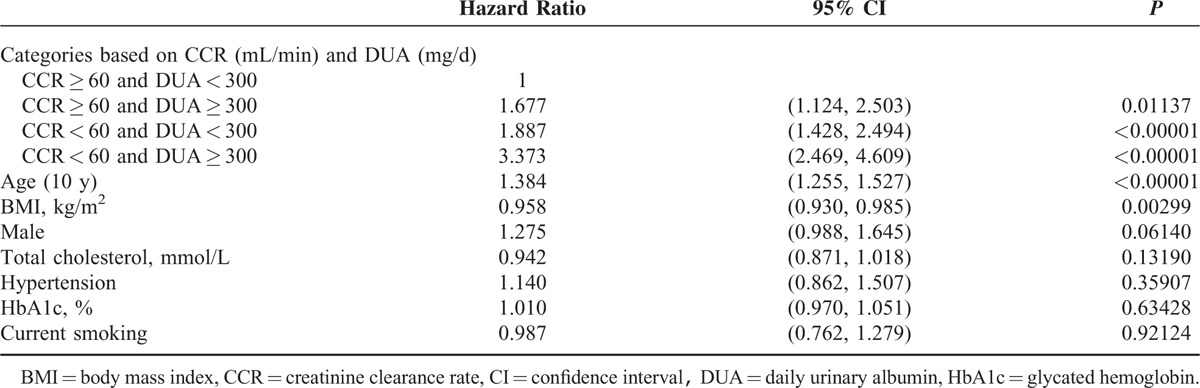
Effects of Creatinine Clearance Rate and Daily Urinary Albumin on Total Mortality in Cox Regression Analysis

**TABLE 6:**
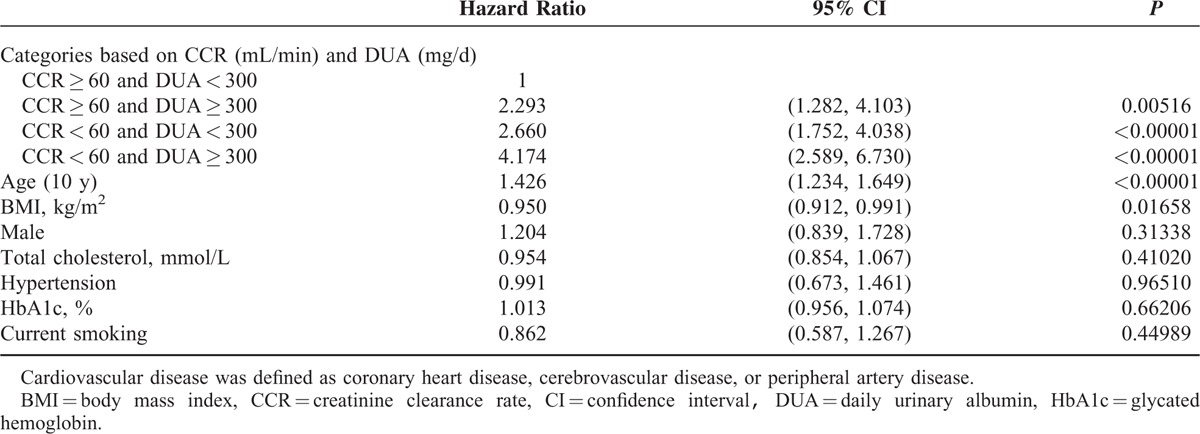
Effects of Creatinine Clearance Rate and Daily Urinary Albumin on Cardiovascular Mortality in Cox Regression Analysis

## DISCUSSION

In the present study, we found a joint effect of albuminuria and CKD on mortality in diabetic patients. That is, the risk of total mortality was highest in diabetic inpatients with both CKD and albuminuria. There was a high mortality rate in our cohort of diabetic inpatients after discharge; more than 55% of patients died during follow-up (median follow-up time of 6.5 years). As the 1-year mortality rate in diabetic patients hospitalized for hyperglycemia in the United States is reportedly around 17%,^16^ early awareness of diabetic nephropathy is important. Our results are similar to previous studies that indicated that albuminuria is significantly associated with mortality in all CKD stages,^18–20^ and that late CKD stages and albuminuria have an additive impact on mortality in diabetic, as well as nondiabetic, subjects.^21–23^ To the best of our knowledge, this is the first study showing that CCR and DUA determined from a 24-hour urine collection in diabetic inpatients are predictive of patient mortality.

It has been reported that albuminuria is not rare in the diabetic population, especially in those of Asian origin.^24^ In the present study, the proportion of subjects with albuminuria was 28% in inpatients with a mean diabetic duration of 9.6 years. The prevalence of albuminuria was higher than the 12%, which was reported in the Asian population who had had diabetes for a mean duration of 7.1 years in the Developing Education on Microalbuminuria for Awareness of Renal and Cardiovascular Risk in Diabetes (DEMAND) study.^24^ The renal function impairment might not reflect the presence of albuminuria. Macisaac et al^25^ demonstrated that only 26% of diabetic patients with CKD had macroalbuminuria (>200 μg/min). It is notable that typical pathological findings of diabetic nephropathy and rapid decline of renal function are seen with macroalbuminuria, rather than normoalbuminuria (<20 μg/min), in diabetic patients with CKD.^26,27^ Our findings support screening for both albuminuria and the CKD stage in diabetic inpatients.

With regard to albuminuria, we found that DUA has a similar predictive ability for patient mortality to ACR. Therefore, as ACR is more convenient than DUA, which requires 24-hour urine collection, ACR remains a good indicator for diabetic complications in clinical practice.^5,6^ On the other hand, although the association between eGFR and mortality has been well investigated,^13^ we found that determination of CKD stage using CCR was more sensitive for predicting mortality than eGFR calculated by the MDRD formula. Furthermore, in diabetic inpatients, we identified low BMI, female gender, low blood pressure, and low serum creatinine as independent factors for a higher eGFR value compared with CCR. In particular, we found that low BMI was the strongest contributor to an overestimated renal function using MDRD formula in both male and female diabetic inpatients. Similarly, a previous study showed an overestimated prevalence of CKD based on an MDRD calculated eGFR in an obese population.^28^ Since a low BMI may lead to an overestimation of eGFR, we suggest using 24-hour urine collection for determination of eGFR to prevent underestimation of CKD stage in diabetic inpatients.

In addition to CKD and albuminuria, we found that a low BMI was an independent factor for predicting mortality. The obesity paradox has been reported in type 2 diabetic patients, and showed a lower mortality rate in overweight subjects compared with that in slimmer ones.^29,30^ In particular, the subjects included in our study had a mean BMI of 23.9 ± 4.2 kg/m^2^, and only 16.5% of subjects had obesity (≥27 kg/m^2^). Therefore, the inverse association between BMI and mortality rate may have been a consequence of the large number of patients with a low or normal BMI. However, the BMI does not accurately reflect the visceral fat component, especially for diabetic inpatients. Indeed, the BMI may have been underestimated in our patient cohort due to dehydration or protein consumption during hyperglycemia, or overestimated due to edema caused by albuminuria.

There are limitations in the present study. First, although diabetic patients were hospitalized due to poor glucose control, the underlying causes were varied. Second, while the 24-hour urine collection was usually arranged when the patient was in a stable condition during hospitalization, there might still be day-to-day variation in the patient's creatinine and albumin concentrations due to activity and/or dehydration. Indeed, hyperglycemia can be accompanied with dehydration or muscle wasting, which could influence the CCR on a daily basis. In this study, we did not eliminate the possibility of day-to-day variation on CCR. On the other hand, even though urinary albumin concentrations can vary with daily activity, previous studies confirm that using a spot urine ACR is an accurate marker of DUA, and their agreement should not be affected by daily variations.^31^ Third, it has been suggested that urine samples for determining the ACR should be collected early in the morning.^6,31^ However, we calculated the ACR from the mean concentration of albumin and creatinine in a 24-hour urine sample. Therefore, our results showing no difference between the DUA and ACR values (ie, a null hypothesis finding between DUA and ACR in the Bland–Altman plot) might have occurred because both values were determined from the same sample. Fourth, we only selected the MDRD formula to estimate GFR, as suggested by the National Kidney Foundation-Kidney Disease Outcomes Quality Initiative (NKF-KDOQI) and broadly applied in Taiwan (including the Taiwan National Database).^32,33^ However, there are several other formulas to estimate GFR, such as the Chronic Kidney Disease Epidemiology Collaboration (CDK-EPI) equation, that have been developed for various patient conditions and may provide a more accurate prediction of patient mortality.^34,35^ Moreover, our findings might only apply to diabetic inpatients, and therefore, further studies are required to confirm the impact of CCR and DUA on predicting mortality in outpatients and the general population.

In conclusion, we found that diabetic nephropathy is an important predictor for mortality in diabetic inpatients after discharge, and albuminuria has an additive contribution on mortality in diabetic inpatients with a lower CCR. Moreover, DUA provides a similar predictive ability on mortality to ACR; however, CCR is more sensitive in predicting mortality than eGFR based on MDRA formula. Finally, we showed that low BMI was associated with a higher eGFR value than CCR in the same diabetic inpatient. Due to the high mortality rate of diabetic inpatients after discharge, further investigation into the contributing factors and preventive strategies for diabetic nephropathy is warranted.
